# Absolute Leukocyte Telomere Length in HIV-Infected and Uninfected Individuals: Evidence of Accelerated Cell Senescence in HIV-Associated Chronic Obstructive Pulmonary Disease

**DOI:** 10.1371/journal.pone.0124426

**Published:** 2015-04-17

**Authors:** Joseph C. Y. Liu, Janice M. Leung, David A. Ngan, Negar F. Nashta, Silvia Guillemi, Marianne Harris, Viviane D. Lima, Soo-Jung Um, Yuexin Li, Sheena Tam, Tawimas Shaipanich, Rekha Raju, Cameron Hague, Jonathon A. Leipsic, Jean Bourbeau, Wan C. Tan, P. Richard Harrigan, Don D. Sin, Julio Montaner, S. F. Paul Man

**Affiliations:** 1 Centre for Heart Lung Innovation, Vancouver, BC, Canada; 2 AIDS Research Program, St. Paul’s Hospital, Vancouver, BC, Canada; 3 Department of Family Medicine, Faculty of Medicine, University of British Columbia, Vancouver, Canada; 4 Division of HIV/AIDS, Department of Medicine, University of British Columbia, Vancouver, Canada; 5 BC Centre for Excellence in HIV/AIDS, St. Paul’s Hospital, Vancouver, BC, Canada; 6 Division of Pulmonary and Critical Care Medicine, Department of Medicine, Dong-A University, Busan, South Korea; 7 UBC Department of Medicine and Division of Respiratory Medicine, St. Paul’s Hospital, Vancouver, BC, Canada; 8 Department of Radiology and Diagnostic Imaging, St. Paul’s Hospital, Vancouver, BC, Canada; 9 Respiratory Epidemiology and Clinical Research Unit, Montreal Chest Institute, McGill University, Montreal, Quebec, Canada; University of Pittsburgh, UNITED STATES

## Abstract

Combination antiretroviral therapy (cART) has extended the longevity of human immunodeficiency virus (HIV)-infected individuals. However, this has resulted in greater awareness of age-associated diseases such as chronic obstructive pulmonary disease (COPD). Accelerated cellular senescence may be responsible, but its magnitude as measured by leukocyte telomere length is unknown and its relationship to HIV-associated COPD has not yet been established. We measured absolute telomere length (aTL) in peripheral leukocytes from 231 HIV-infected adults. Comparisons were made to 691 HIV-uninfected individuals from a population-based sample. Subject quartiles of aTL were assessed for relationships with measures of HIV disease severity, airflow obstruction, and emphysema severity on computed tomographic (CT) imaging. Multivariable regression models identified factors associated with shortened aTL. Compared to HIV-uninfected subjects, the mean aTL in HIV-infected patients was markedly shorter by 27 kbp/genome (p<0.001); however, the slopes of aTL vs. age were not different (p=0.469). Patients with longer known durations of HIV infection (p=0.019) and lower nadir CD4 cell counts (p=0.023) had shorter aTL. Shorter aTL were also associated with older age (p=0.026), smoking (p=0.005), reduced forced expiratory volume in one second (p=0.030), and worse CT emphysema severity score (p=0.049). HIV-infected subjects demonstrate advanced cellular aging, yet in a cART-treated cohort, the relationship between aTL and age appears no different from that of HIV-uninfected subjects.

## Introduction

Combination antiretroviral therapy (cART) has lengthened the life expectancy of patients living with human immunodeficiency virus (HIV) [1]. Fewer HIV-infected individuals die from opportunistic infections and AIDS-related cancers [2]; instead, they now face chronic conditions normally associated with old age. Recently, studies have identified an increased prevalence of chronic obstructive pulmonary disease (COPD) within HIV-infected populations even after adjustment for age and smoking [3–5]. Clinical manifestations of COPD in the HIV population appear to be far more severe [6] and develop earlier than in HIV-uninfected individuals [4].

Disease models of accelerated cellular senescence apply well to HIV [7] and may explain the propensity towards age-associated conditions. In HIV, leukocyte telomere lengths, a marker of cellular aging, are comparatively shorter when measured against uninfected patients [8–10]. Telomeres are structures found at the ends of chromosomes, made up of repetitive TTAGGG DNA sequences and responsible for protecting genomic integrity. While telomere shortening occurs with each cell cycle, once a critical length is reached cell cycle arrest or apoptosis ensues [11]. Immune activation and microbial translocation are thought to drive telomere shortening in HIV [12], but the magnitude of telomere shortening that occurs upon infection with HIV remains unknown.

We postulate that accelerated cellular aging is a major driver for HIV-associated COPD and one that warrants further investigation. Although recent literature in HIV-uninfected cohorts has shown that telomere length is associated with COPD [13–15] and can predict mortality in COPD [16], whether telomere shortening is associated with more severe COPD specifically in HIV-infected populations is currently unknown.

The purpose of this study is twofold. First, we aim to quantify the magnitude of telomere shortening that occurs with HIV using a novel technique that measures absolute rather than relative telomere length [17–19]. This method, which standardizes quantitative polymerase chain reaction (qPCR) telomere length measurements, allows for cross-experimental and cross-study comparisons and may in future provide a universal high-throughput platform with which to measure telomere attrition. Accordingly, we measured absolute leukocyte telomere length (aTL) in a cohort of HIV-infected patients as well as in a large HIV-uninfected cohort. Second, we aim to investigate whether measures of COPD severity, including spirometry and extent of emphysema on lung imaging, are associated with aTL in the setting of HIV.

## Materials and Methods

### Study Populations

Participants were derived from a cohort of HIV-infected patients at St. Paul’s Hospital in Vancouver, British Columbia, Canada. The cohort was assembled as part of a prospective observational study of HIV-associated lung disease with enrollment taking place between August 2009 and June 2013 [6]. All cohort participants had serologically documented HIV-1 infection and were aged 19 years or older; COPD was not a specific inclusion criterion. Following informed consent, demographic data and clinical history were collected, and each participant had spirometry performed according to the American Thoracic Society/European Respiratory Society recommendations [20]. Individual forced expiratory volume in one second (FEV1) measurements were expressed as a percentage of predicted (FEV1%Pred) using the NHANES III reference equation [21]. HIV-related clinical information, including laboratory data and cART history, were obtained through a linkage with the British Columbia Centre for Excellence in HIV/AIDS Drug Treatment Program database. The cut-off for successful viral suppression was determined to be <40 copies/mL [22]. Comorbidities such as hepatitis C, asthma, and cardiomyopathy were determined from the subject’s medical records. Moderate alcohol use was defined as the intake of ≥0.22 fl oz of alcohol per day [23]. Only the cross-sectional data derived from the participant’s first study visit were included in this analysis.

An HIV-uninfected control sample was derived from the Canadian Cohort of Obstructive Lung Disease (CanCOLD) study. CanCOLD is an ongoing, prospective longitudinal cohort study evaluating COPD progression. The cohort is originally derived from a larger cross-sectional population-based cohort (COLD) that evaluated COPD prevalence in 5,176 adults in nine Canadian cities (Vancouver, Calgary, Saskatoon, Montreal, Toronto, Quebec City, Ottawa, Kingston, and Halifax). These COLD participants were recruited by random digit dialing using census data from Statistics Canada with a mean participation rate of 75% [24]. From these participants, 1,140 non-COPD and COPD patients were enrolled for longitudinal follow-up to form the CanCOLD study cohort. The derivation of the longitudinal cohort for follow-up has been described in detail in a previous publication [25]. Enrollment in CanCOLD occurred between 2009 and 2013. Demographic data including age, sex, and smoking status and duration were available for this cohort, as were pre-bronchodilator spirometry measurements including FEV1 and FEV1%Pred, forced vital capacity (FVC), and FVC percent predicted (FVC %Pred). The data from the initial visit of the longitudinal cohort was used in this study. These data were collected at the same visit as the collection of venous blood samples. At the time of the current study, venous blood for telomere measurement was available in 691 consecutive individuals. The samples from the subjects with HIV and from the non-HIV population-based CanCOLD cohort were collected, processed and stored using the same protocol and analyzed in the same laboratory.

### Measurement of aTL in Peripheral Leukocytes

Genomic DNA from peripheral leukocytes in HIV-infected participants and CanCOLD participants was harvested using the Qiagen DNeasy Blood & Tissue Kit (Qiagen, Venlo, the Netherlands). Effort was made to ensure samples had undergone only one freeze-thaw cycle before the extraction process. aTL was measured by quantitative PCR consistent with methods outlined by O’Callaghan and Fenech [17]. In brief, standard curves were generated from known quantities of synthesized oligomers of telomere (*TEL*) DNA [(TTAGGG)14] and single copy gene (*36B4*) DNA [CAGCAAGTGGGAAGGTGTAATCCGTCTCCACAGACAAGGCCAGGACTCGTTTGTACCCGTTGATGATAGAATGGG] (Sigma-Aldrich, St. Louis, MO). Sample telomere DNA length was then assessed based on the ratio of telomere DNA length to *36B4* DNA length as obtained from their respective standard curves. DNA from a short telomere cell line (*HEK293*) and a long telomere cell line (*K562*) (ATCC, Manassas, VA) were used as inter-experimental plate controls [26]. The telomere lengths measured using this method reflect an average length across the population of cells included in the sample. Samples were run in triplicate using the ABI ViiA 7 Real Time PCR System (Applied Biosystems, Foster City, CA). Both HIV and non-HIV samples were concurrently run on the same plate to avoid batch effects.

### Measurement of HIV RNA, CD4 Cell Count, and Inflammatory Biomarkers

Blood samples were collected after an overnight fast using standard venipuncture methods, separated into their various components, and assayed for CD4 cell count and plasma HIV-1 RNA level (Roche Amplicor Ultrasensitive Assay and Roche Taqman Ultrasensitive Assay, Laval, Quebec, Canada) in the laboratory facilities of St. Paul’s Hospital. Plasma samples which had been stored at -80°C were thawed once for the measurements of C-reactive protein (CRP) and interleukin-6 (IL-6). All biomarker assays were measured in duplicate and the mean value of the duplicates for each sample was used for statistical analysis.

### Computed Tomography (CT) Scans

Within one year of study enrollment, chest CT images were acquired in an unselected subset of the HIV-infected cohort, using a 64 detector CT scanner (VCT XT and Discovery HD 750 GE Healthcare, Waukasha, WI) under a modified clinical low effective dose protocol (1·0-mm slice thickness, 120 kVp, 215 mA, 0·6-second rotation time and pitch of 1·5, reconstructed using both high and intermediate spatial frequency reconstruction kernels). Two highly experienced radiologists (JAL and RR), blinded to spirometry and laboratory data, interpreted the CT images together to achieve consensus. Emphysema severity was quantified based on a modified method of Kazerooni *et al*. [27], also employed in the COPDGene study [28]. Individual scores for each of the five lobes plus the lingula were assessed as follows: 0 = absence of emphysema, 1 = 1–25% emphysema, 2 = 26–50% emphysema, 3 = 51–75% emphysema, and 4 = 76–100% emphysema. A total score was obtained by the summation of the scores of the five lobes and lingula (see S1 Fig for representative images). A total score of 0 indicated the absence of emphysema; a score of 1–2 indicated mild emphysema; 3–4 denoted moderate emphysema; and >4 indicated severe emphysema.

### Statistical Analysis

We first divided the HIV cohort into quartiles of aTL. The baseline characteristics across the quartiles were compared using the Jonchkeere-Terpstra trend test for continuous variables and the Cochran-Armitage test for dichotomous variables. Biomarkers were log-transformed to achieve normality. Collinearity, normality, and heteroscedascity were assessed to validate the linear regression models. To compare the respective aTL relationships with age between HIV and CanCOLD populations, a linear regression model was made using telomere length as a continuous dependent variable. Because of the availability of data on the CanCOLD subjects, only age, sex, smoking status, BMI, and FEV1%Pred were included as covariates.

To create the multivariable linear regression models with aTL as the output, important variables from univariate analyses (p<0.15) associated with aTL were included as candidates for the multivariable model. Then, a backward stepwise procedure was employed to build the multivariable linear regression model. The selection of variables was based on two criteria: Akaike Information Criterion (AIC) and Type I p-values. At each step during the backward stepwise multivariate analysis, the AIC value and the Type I p-value of each variable were recorded and the variable with the highest Type I p-value was dropped until we obtained the lowest AIC. These models were first created to examine the HIV only population to determine which factors may contribute to telomere shortening within HIV and secondly to the combined HIV and non-HIV population to determine the relative contribution of HIV itself to telomere shortening. To examine the robustness of the HIV only statistical model employed, secondary ordinal logistic regression models were performed using the quartiles of aTL as the output; however, since the results were similar to the linear regression models, only the results from the linear regression models are reported here.

All analyses were performed using JMP statistical software (version 10.0; SAS Institute, Cary, NC) and two-sided p-values <0.05 were considered significant.

### Ethics/Informed Consent

This study was conducted in accordance with the amended Declaration of Helsinki and received approval from the UBC Providence Health Care ethics review committee (No.H11-02713). All subjects provided written informed consent.

## Results

### Subjects

**Fig 1:**
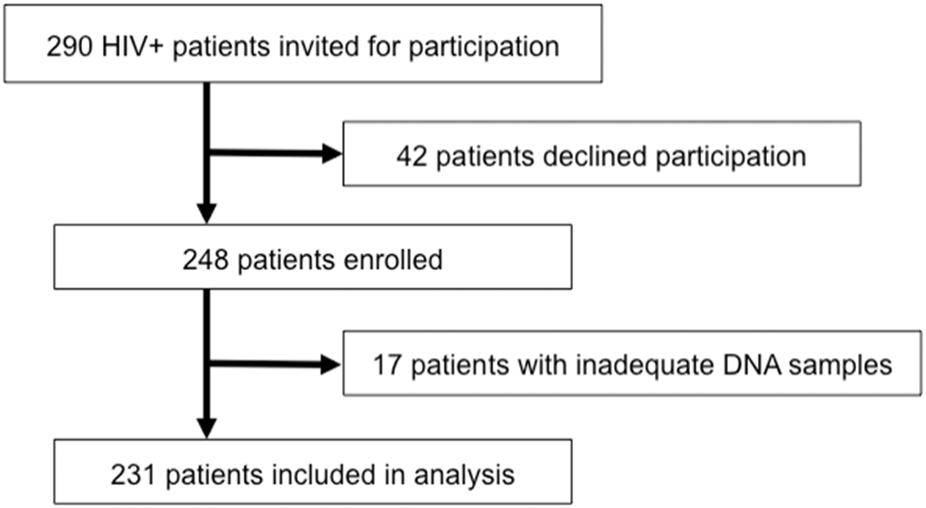
Study Population. Flow chart representing patients included and excluded in the A) HIV cohort and B) CanCOLD cohort.

Fig 1 represents the flow chart for study inclusion (Fig 1A for the HIV-infected cohort and Fig 1B for the CanCOLD cohort). A total of 231 individuals formed the HIV-infected cohort. Baseline demographic and respiratory-related data for these subjects are shown in Table 1. The majority (91%) were male and the mean age was 49.6 years.

**Table 1:** Baseline Characteristics of Study Participants, For All HIV+ Subjects and By Telomere Length Quartiles.

		Telomere Quartiles (kbp/genome)				
Group	All	1^st^ (<110.2)	2^nd^ (110.2–132.4)	3^rd^ (132.5–155.8)	4^th^ (≥155.9)	P-trend
Telomere length (kbp/genome)	131.6 (30.0)	92.7 (11.7)	120.5 (6.1)	144.1 (7.1)	169.8 (11.3)	
No. of patients	231	58	58	58	57	
No. of male patients (% of total)	211 (91%)	55 (95%)	54 (93%)	52 (90%)	50 (88%)	0.135
Age (years)	49.6 (10.2)	51.6 (9.2)	49.8 (10.0)	50.0 (10.8)	47.0 (10.6)	0.026
Body mass index (kg/m²)	25.5 (4.6)	24.6 (4.0)	25.3 (4.7)	25.9 (4.6)	26.6 (4.3)	0.011
Ever smoker	187 (81%)	53 (91%)	48 (83%)	44 (76%)	42 (73%)	0.005
Current Smoker	128 (55%)	30 (52%)	34 (59%)	35 (60%)	29 (51%)	0.984
Smoking, pack-years†	29.7 (20.2)	28.2 (17.6)	30.5 (20.0)	29.9 (21.8)	23.8 (14.5)	0·263
Ever marijuana smoker	140 (61%)	40 (69%)	40 (69%)	31 (53%)	29 (51%)	0.015
Ever crack smoker	70 (30%)	21 (36%)	13 (22%)	20 (34%)	16 (28%)	0.649
Ever IDU	81 (35%)	19 (33%)	24 (41%)	19 (33%)	19 (33%)	0.888
Moderate-heavy alcohol use	161 (70%)	45 (78%)	39 (67%)	41 (71%)	36 (63%)	0.142
CRP (ug/mL)	2.5 (2.7)	2.5 (2.8)	2.1 (2.3)	2.2 (2.6)	2.8 (2.9)	0.711
IL-6 (pg/mL)	2.5 (2.5)	2.6 (2.4)	2.2 (2.3)	2.3 (2.4)	2.8 (2.8)	0.626
*Co-morbidities*						
Hepatitis C	51 (22%)	14 (24%)	14 (24%)	10 (17%)	13 (23%)	0.652
Asthma	50 (23%)	10 (18%)	8 (15%)	17 (33%)	15 (27%)	0.083

Mean (SD) values are given for normally distributed variables, while dichotomous data are given as counts (% of total).

Abbreviation definition: IDU = injection drug use; CRP = C-reactive protein; IL-6 = interleukin-6.

^†^for current and ex-smokers.

The HIV-infected cohort was segregated into quartiles based on aTL. Significant trends included older age (p = 0.026) and lower body mass index (BMI) (p = 0.011) in association with shorter aTL. Just over half of study participants were current smokers; participants with shorter aTL were more likely to have ever smoked (p = 0.005). Prior marijuana smoking (p = 0.015) was also associated with shorter aTL. Levels of inflammatory markers, CRP and IL-6, did not differ across quartiles of aTL (p = 0.711 and 0.626, respectively). Only two patients carried a diagnosis of cardiomyopathy and there were no cases of concurrent pulmonary hypertension.

**Fig 2:**
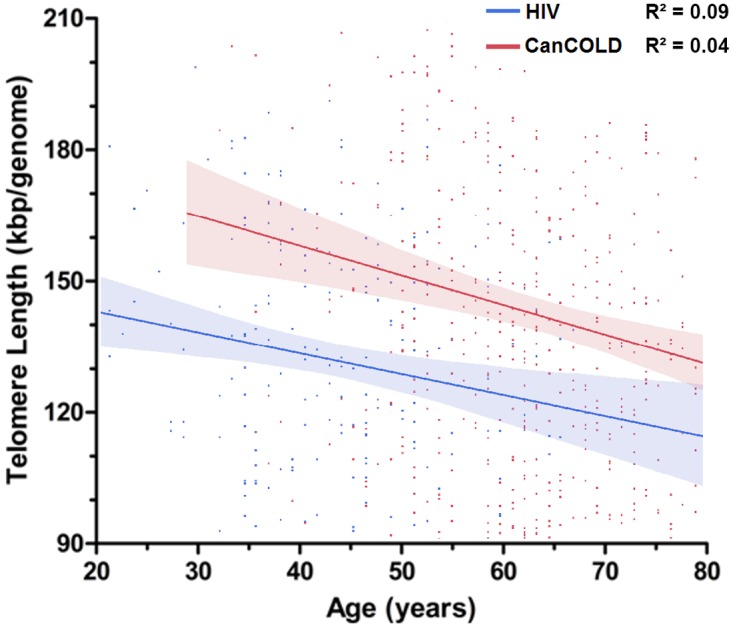
Absolute leukocyte telomere length by age for HIV-infected and non-HIV-infected individuals. When HIV-infected individuals (blue) are compared to non-HIV-infected individuals (red) from the CanCOLD cohort, significant differences in telomere length are seen. The respective slopes of aTL vs. age do not differ significantly between CanCOLD and HIV populations (p = 0.469). Solid lines represent the regression line; shaded areas represent the 95% confidence interval; the analysis has been adjusted for sex, BMI, smoking history and FEV1%Pred.

**Fig 3:**
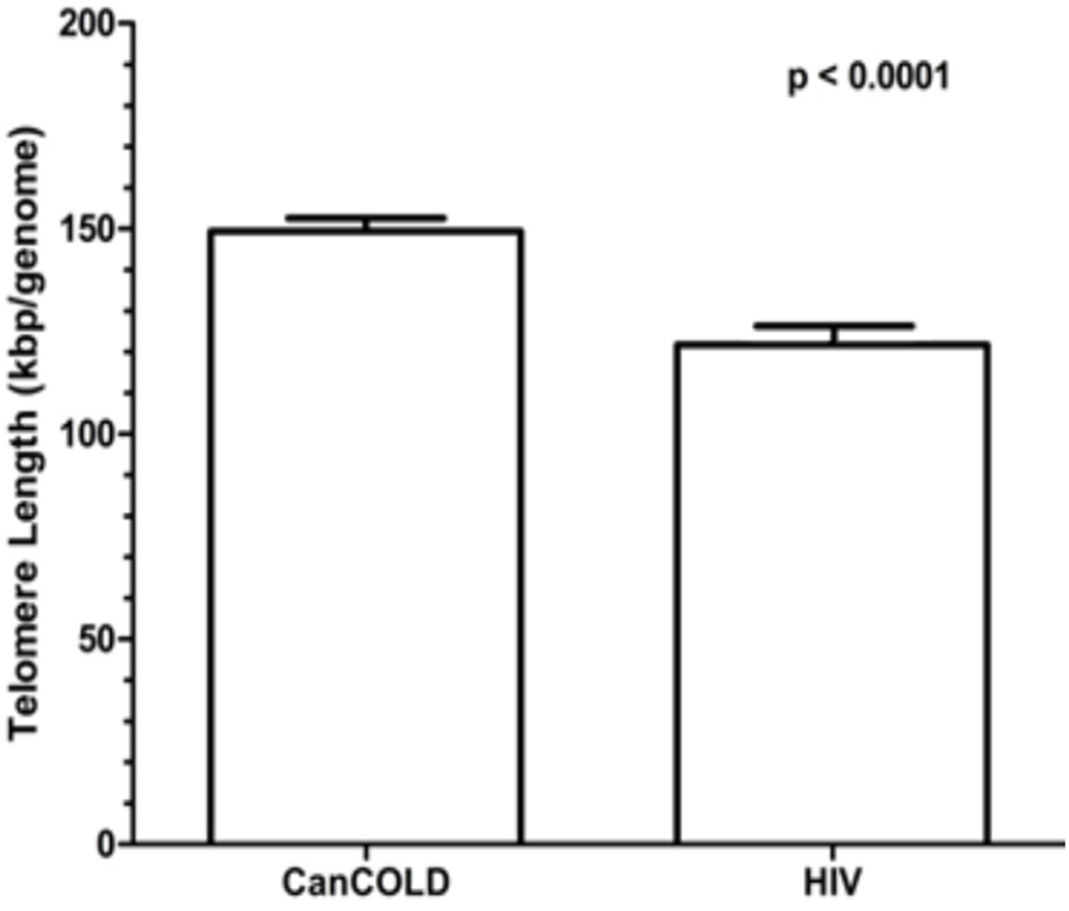
Absolute leukocyte telomere length differences between HIV-infected and non-HIV-infected individuals. Differences in the mean aTL between the CanCOLD and HIV populations after adjustment for age, gender, BMI, smoking pack-years, and FEV1%Pred. The aTL of the CanCOLD population was significantly different from that of the HIV population (p<0.0001). Error bars represent the standard error.

Additionally, aTL were measured in 691 HIV-uninfected individuals from the CanCOLD cohort (see Table 2 for demographic information). After adjusting for sex, BMI, smoking, and FEV1%Pred, there was a significant negative correlation between telomere length and age, in both the CanCOLD and HIV groups (R^2^ = 0.04, p<0.001 and R^2^ = 0.09, p = 0.030, respectively) (Fig 2). The slopes of aTL vs. age were similar between the two groups (p = 0.469) with both groups sharing a common slope of -0.713±0.155 kbp/genome/year (p<0.001). The difference in mean aTL between the CanCOLD and HIV populations (after adjustment for age, gender, BMI, smoking pack-years, and FEV1%Pred) is shown in Fig 3. The mean±SEM aTL for the CanCOLD and HIV populations were 150±3 and 123±4 kbp/genome, respectively (p<0.0001).

**Table 2:** Characteristics of CanCOLD and HIV Subjects.

Characteristic	CanCOLD	HIV	P-value
No. of subjects	691	231	
Men (% of total)	345 (40%)	211 (91%)	<0.001
Age (years)	66.4 (9.6)	49.3 (10.1)	<0.001
Body mass index (kg/m²)	27.7 (5.3)	25.5 (4.6)	<0.001
Ever smokers	438 (63%)	187 (81%)	<0.001
FEV1% predicted	89.6 (19.8)	84.3 (22.1)	0.001
FEV1/FVC ratio (%)	69.0 (10.7)	69.4 (17.4)	0.016

Mean (SD) values are given for continuous variables, while dichotomous data are given as counts (% of total).

### Telomere Length and HIV-Specific Variables

Associations between aTL, HIV-specific variables, and AIDS-defining illnesses are shown in Table 3. Almost all study participants (94%) were on cART at the time of study enrollment with 156 (70%) patients achieving undetectable viral loads (<40 copies/mL). Longer time from HIV diagnosis to enrollment, an estimate of the duration of HIV infection, was associated with the shortest aTL (p = 0.019). In addition, lower nadir CD4 cell counts were also associated with shorter aTL (p = 0.023), while plasma viral loads >100,000 copies/mL at the time of HIV diagnosis did not show a significant relationship. Although current CD4 cell counts tended to be lower in individuals with the shortest aTL, this did not achieve statistical significance (p = 0.065). Twenty-nine (13%) had prior *Pneumocystis jirovecii* pneumonia (PJP). However, a history of PJP was not statistically significant when comparing across aTL quartiles (p = 0.098).

**Table 3:** HIV-Related Clinical and Laboratory Data, For All HIV+ Subjects and By Telomere Length Quartiles.

		Telomere Quartiles (kbp/genome)				
Group	All	1^st^ (<110.2)	2^nd^ (110.2–132.4)	3^rd^ (132.5–155.8)	4^th^ (≥155.9)	P-trend
Telomere length (kbp/genome)	131.6 (30.0)	92.7 (11.7)	120.5 (6.1)	144.1 (7.1)	169.8 (11.3)	
No. of patients	231	58	58	58	57	
Time from HIV diagnosis to enrollment (months)	143 (91)	149 (93)	158 (97)	154 (87)	108 (81)	0.019
*Current cART use*	216 (94%)	55 (95%)	53 (91%)	58 (100%)	50 (88%)	0.389
NNRTI	64 (27%)	19 (33%)	16 (28%)	15 (26%)	14 (25%)	0.318
NRTI	204 (88%)	52 (90%)	50 (86%)	55 (95%)	47 (82%)	0.500
PI	132 (57%)	35 (60%)	30 (52%)	37 (64%)	30 (53%)	0.708
*At time of HIV diagnosis*						
Plasma viral load >100,000 HIV RNA copies/mL	120 (56%)	34 (63%)	28 (52%)	29 (57%)	29 (54%)	0.450
Nadir CD4 (cells/μL)	209 (177)	184 (168)	182 (160)	222 (196)	246 (167)	0.023
Nadir CD4 ≤350 cells/μL	179 (85%)	50 (93%)	47 (87%)	44 (86%)	38 (73%)	0.007
*At study enrollment*						
Plasma viral load <40 HIV RNA copies/mL	156 (70%)	39 (70%)	40 (71%)	42 (75%)	35 (64%)	0.605
CD4 (cells/μL)	529 (245)	522 (258)	481 (235)	543 (198)	576 (243)	0.065
CD4 ≤350 cells/μL	51 (22%)	15 (26%)	15 (26%)	10 (18%)	11 (19%)	0.253
Prior respiratory infection	98 (45%)	27 (48%)	22 (42%)	29 (55%)	20 (36%)	0.445
PJP Infection	29 (13%)	9 (16%)	9 (16%)	8 (14%)	3 (5%)	0.098
Candidiasis of esophagus (% total)	16 (7%)	5 (9%)	3 (5%)	4 (7%)	4 (7%)	0.835
Herpes Simplex (% total)	167 (72%)	13 (22%)	20 (34%)	16 (28%)	15 (26%)	0.852

Abbreviation definition: cART = combination antiretroviral therapy; PI = protease inhibitor; NNRTI = non-nucleoside reverse transcriptase inhibitor; NRTI = nucleoside reverse transcriptase inhibitor; PJP = *Pneumocystis jirovecii* pneumonia

### Telomere Length and COPD

Respiratory variables for the HIV cohort are shown in Table 4. Lower FEV1, FEV1%Pred, and FEV1/FVC showed a significant trend with shorter aTL (p = 0.030, 0.029, and 0.037, respectively), while FVC and FVC %Pred did not. Among the 109 patients who underwent chest CT scanning, total CT emphysema scores ranged from 0 to 19 (S1 Table is provided to show demographic differences between those who underwent CT scanning and those who did not). A greater proportion of individuals with the shortest aTL had severe emphysema (CT emphysema score >4) (p = 0.049).

**Table 4:** Respiratory Related Variables, For All HIV+ Subjects and By Telomere Length Quartiles.

		Telomere Quartiles (kbp/genome)				
Group	All	1^st^ (<110.2)	2^nd^ (110.2–132.4)	3^rd^ (132.5–155.8)	4^th^ (≥155.9)	P-trend
Telomere length (kbp/genome)	131.6 (30.0)	92.7 (11.7)	120.5 (6.1)	144.1 (7.1)	169.8 (11.3)	
No. of patients	231	58	58	58	57	
FEV1 (Liters)	3.1 (0.9)	2.9 (0.9)	3.2 (1.0)	3.1 (0.9)	3.4 (0.9)	0.030
FEV1%Pred	84.3 (22.1)	79.0 (23.3)	84.9 (25.2)	83.6 (20.6)	89.5 (19.2)	0.029
FVC (Liters)	4.4 (1.0)	4.2 (0.9)	4.5 (1.0)	4.2 (1.1)	4.5 (1.1)	0.377
FVC %Pred	91.8 (17.4)	90.0 (17.1)	93.1 (17.7)	89.0 (17.0)	94.6 (17.6)	0.207
FEV1/FVC (%)	69.4 (17.4)	67.8 (15.0)	69.0 (17.4)	71.5 (14.9)	73.0 (13.3)	0.037
Total CT Emphysema Score	2.7 (4.1)	3.8 (4.7)	3.9 (5.5)	1.2 (2.1)	2.0 (2.6)	0.121
CT Emphysema Score > 4	30 (13%)	12 (21%)	8 (14%)	5 (9%)	5 (9%)	0.049

Abbreviation definitions: FEV1 = forced expiratory volume in 1 second; FEV1%Pred = forced expiratory volume in 1 second percent predicted; FVC = forced vital capacity; FVC %Pred = forced vital capacity percent predicted; CT = computed tomography.

### Multivariable Analyses

Multivariable analyses are shown in Tables 5 and 6. Within the HIV population only (Table 5), significant predictors of shorter aTL in a multivariate linear regression model included older age (p = 0.010), a history of smoking (p = 0.010), lower BMI (p = 0.034), and having a nadir CD4 cell count ≤350/μL (p = 0.018). Prior PJP infection, although included in the model, was not associated with aTL (p = 0.065).

**Table 5:** Multivariable Regression Model: HIV Only.

Linear Regression Model, HIV Only (Outcome: Telomere Length, Continuous)					
	Unadjusted		Multivariable Model: R² = 0.14; p <0.01		
	Effect Estimate (SE)*	P-value	β (SE)	Standardized β	P-value
Ever smoker	-0.021 (0.007)	0.002	-6.418 (2.466)	-0.171	0.010
Age (years)	-0.069 (0.022)	0.002	-0.507 (0.194)	-0.175	0.010
Nadir CD4 ≤350 cells/μL	-0.020 (0.006)	0.003	-6.488 (2.726)	-0.158	0.018
Body Mass Index (kg/m²)	0.023 (0.010)	0.018	0.916 (0.429)	0.140	0.034

Abbreviations: SE = standard error

**Table 6:** Multivariable Regression Model: HIV and Non-HIV.

Linear Regression Model, HIV and Non-HIV (Outcome: Telomere Length, Continuous)					
	Unadjusted		Multivariable Model: R² = 0.05; p <0.01		
	Effect Estimate (SE)*	P-value	β (SE)	Standardized β	P-value
HIV Status (Infected)	-0.005 (0.002)	0.004	-10.406 (2.211)	-0.193	<0.001
Age (years)	-0.016 (0.009)	0.058	-0.755 (0.155)	-0.197	<0.001
Ever Smoker	-0.005 (0.002)	<0.001	-4.769 (1.692)	-0.095	0.005
FEV1%Pred	0.037 (0.015)	0.012	0.099 (0.076)	0.044	0.190

Abbreviations: SE = standard error; FEV1: forced expiratory volume in 1 second

In a multivariable linear regression analysis evaluating characteristics associated with shorter aTL for both the HIV and CanCOLD cohorts combined (Table 6), older age (p<0.001), a history of smoking (p = 0.006), the presence of HIV (p = 0.037) remained significant predictors of shorter aTL. FEV1%Pred did not remain a significant predictor of shorter aTL in the final model.

## Discussion

In this study, we have quantified aTL shortening in an HIV-infected population, identifying a mean telomere length difference of approximately 27 kbp/genome when compared to a general population sample. Despite this considerable shortening, our population of cART-treated HIV-infected patients demonstrated a slope of aTL vs. age that was similar to that of an HIV-uninfected cohort. We speculate that aTL may have acutely shortened prior to study enrollment, perhaps at the time of acute HIV infection or during periods of the most severe immunosuppression. Indeed, low nadir CD4 cell counts appear to place patients at highest risk for shortened aTL. Even in the absence of overt opportunistic infections, immunosuppression may be sufficient to cause accelerated aging, as we did not see an equivalent relationship between the presence of AIDS-defining conditions like PJP and aTL. Since the vast majority of our patients were receiving cART with adequate viral suppression and CD4 cell count normalization, the rate of telomere shortening may have stabilized. In fact, the findings of our study lead one to speculate that the early initiation of cART could abrogate the accelerated aging process and potentially avert adverse consequences such as COPD. Future studies assessing the impact of early cART initiation on the recent epidemic of age-related conditions in HIV would be a welcome addition to the field.

Our findings are in keeping with previous studies that have also demonstrated telomere shortening in HIV when compared to the general population [9,10]. However, we found different characteristics to be significantly associated with shortened aTL in our cohort of patients. Most recently, Zanet *et al*. found that patients with active hepatitis C and a peak HIV viral load ≥100,000 copies/mL were more likely to have short telomeres, two relationships that were not found in our study. As well, Pathai *et al*. found that low current CD4 cell counts were also associated with short aTL, whereas only nadir CD4 cell counts were significantly associated with aTL in our analysis. Differences in study populations may account for these discrepancies, as our population was mostly male as opposed to the predominantly female HIV-infected cohorts previously studied (females are generally believed to have longer telomeres than males [29,30]). In addition, a greater proportion of our patients were on cART in comparison to those in the two previous studies. Nonetheless, our study echoes a consistent theme that greater severity of HIV may be contributing to accelerated cellular aging.

The application of telomere biology to HIV-associated COPD yielded insight that accelerated aging may be an important driver of lung disease in this population, as we demonstrate that aTL is inversely related to lung function. This concurs with a recent study demonstrating that relative telomere length in peripheral blood mononuclear cells is also shortened in HIV patients with airflow obstruction and diffusion capacity impairment [31]. Although peripheral leukocyte aTL reflects a systemic rather than local aging signal, these data suggest that cellular aging might also occur in lung-specific tissue. Recent evidence, for example, points to an excellent correlation between peripheral leukocyte and lung tissue telomere length in patients with α1-antitrypsin deficiency [32]. A similar relationship may exist in HIV-associated COPD, but requires further assessment. Our findings also support the known associations in HIV-uninfected patients between greater severity of airflow obstruction and shorter peripheral leukocyte [13–15] and lung tissue [33] telomere lengths. While HIV may be one trigger for accelerated aging, the combination of HIV and smoking, the latter of which demonstrated a significant association with shortened telomere lengths in our study, may prove to be a particularly potent dual hit to the telomere. Profound telomere shortening as a result may help incite the apoptosis that leads to the early development of COPD.

One of the strengths of our study is the use of a novel technique for measuring telomere length, a qPCR method that measures absolute rather than relative length. Unlike relative telomere length measurements in which quantification is dependent on the type of single copy gene standard chosen by each laboratory, absolute measurements allow for comparison across studies, providing a standardized method while still maintaining high volume capacity. aTL measurements are now becoming the preferred method for studying telomere length [18,19] and in future may be a more useful biomarker of aging given its standardization. The ability of aTL to predict which HIV-infected patients are at greatest risk for developing age-associated conditions such as COPD has yet to be determined, but our study suggests promise for future exploration.

Our study has several limitations, including the fact that we measured aTL in undifferentiated leukocytes. Measurements specifically performed in rapidly proliferating CD8+ T cells may have in fact revealed even shorter telomere lengths. If anything, our measurements are likely to overestimate aTL in our HIV cohort as a result. We did not in addition find any significant association between aTL and CD8+ T cell count or total leukocyte count (data not shown). Furthermore, aTL can only be measured in intact cells, raising the possibility that cellular environments prone to rapid apoptosis may not necessarily be reflected in the measurements obtained. As well, only half of our cohort was able to undergo chest CT scanning, somewhat limiting our ability to assess relationships between emphysema scoring and aTL. Finally, the majority of our HIV-infected cohort was male, so generalization to HIV-infected females may be limited. This is largely due to the fact that as a single-center study we were unable to capture many HIV-infected females who receive their HIV-related care at a different hospital in our city.

In summary, we demonstrate that although HIV infection is associated with a large and quantifiable shortening of telomeres, the declining slope of aTL vs. age may be attenuated in a population treated with cART. The use of a qPCR method to determine aTL may provide future studies in this field a common platform to compare a useful biomarker of aging. Furthermore, within HIV-infected individuals, the presence of COPD is associated with shorter telomeres, supporting an accelerated aging mechanism contributing to the development of COPD in HIV disease. Further studies should evaluate whether early cART initiation with subsequent suppression of HIV viral replication can normalize the rate of telomere attrition and prevent the onset of comorbidities such as COPD.

## Supporting Information

S1 Fig Representative Emphysema Score Images. A. Score 0 (absence of emphysema). B. Score 1 (1–25% emphysema). C. Score 2 (26–50% emphysema). D. Score 3 (51–75% emphysema). E. Score 4 (76–100% emphysema).
(TIF)

S1 Table Characteristics of those who underwent CT and those who did not. (DOCX)
