# Supplementary figures and images for: Absolute Leukocyte Telomere Length in HIV-Infected and Uninfected Individuals: Evidence of Accelerated Cell Senescence in HIV-Associated Chronic Obstructive Pulmonary Disease
Source: PLoS One. 2015 Apr 17;10(4):e0124426. doi: 10.1371/journal.pone.0124426 (PMC4401786; doi:10.1371/journal.pone.0124426)

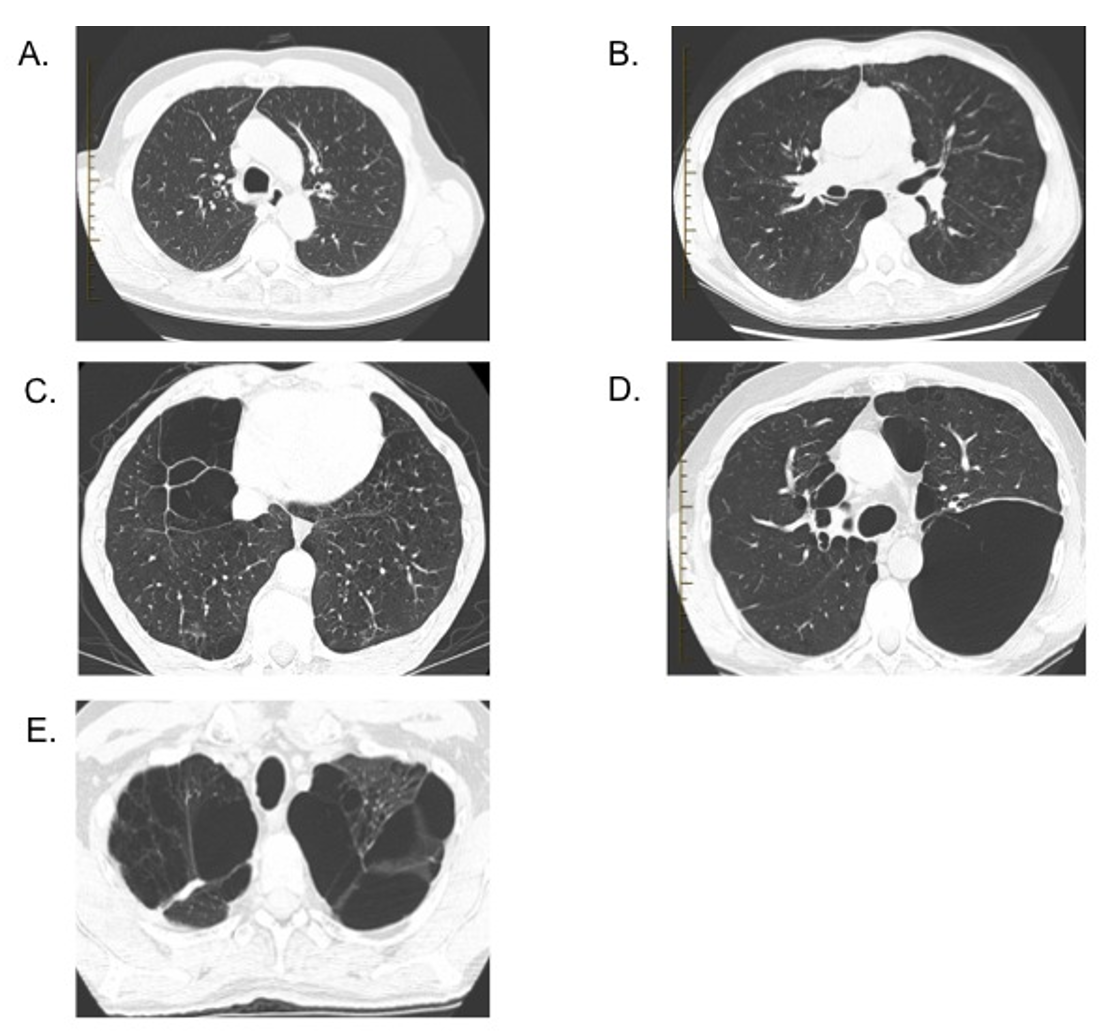

Supplement: S1 Fig — A. Score 0 (absence of emphysema). B. Score 1 (1–25% emphysema). C. Score 2 (26–50% emphysema). D. Score 3 (51–75% emphysema). E. Score 4 (76–100% emphysema). (TIF) [file pone.0124426.s001.tif]
