# Supplementary material for: Absolute Leukocyte Telomere Length in HIV-Infected and Uninfected Individuals: Evidence of Accelerated Cell Senescence in HIV-Associated Chronic Obstructive Pulmonary Disease
Source: PLoS One. 2015 Apr 17;10(4):e0124426. doi: 10.1371/journal.pone.0124426 (PMC4401786; doi:10.1371/journal.pone.0124426)
Supplement: S1 Table — (DOCX) [file pone.0124426.s002.docx]

**S1 Table. Characteristics of those who underwent CT and those who did not.**

| **Characteristic** | **CT** | **No CT** | **P-value** |
| --- | --- | --- | --- |
| No. of subjects | 109 | 122 |  |
| Men (% of total) | 109 (100%) | 102 (84%) | <0.001 |
| Age (years) | 51.3 | 48.2 | 0.021 |
| Body mass index (kg/m²) | 25.6 (4.5) | 25.5 (4.4) | 0.894 |
| Ever smokers | 88 (81%) | 100 (82%) | 0.810 |
| Smoking, pack-years† | 29.3 (18.7) | 27.2 (18.7) | 0.428 |
| FEV1 % predicted | 81.5 (22.7) | 86.7 (21.9) | 0.078 |
| FEV1/FVC ratio (%) | 68.5 (15.1) | 72.0 (15.3) | 0.084 |
| CD4 (cells/µL) | 535 (243) | 526 (230) | 0.781 |
| Plasma viral load <40 HIV RNA copies/mL | 84 (77%) | 72 (63%) | 0.023 |

Mean (SD) values are given for normally distributed variables, while dichotomous data are given as counts (% of total).

†for current and ex-smokers.

Abbreviations: CT – computed tomography scan; FEV1 – forced expiratory volume in 1 second; FVC – forced vital capacity
